# Emotion Regulation, Peer Acceptance and Rejection, and Emotional–Behavioral Problems in School-Aged Children

**DOI:** 10.3390/children12020159

**Published:** 2025-01-28

**Authors:** Nicoletta Salerni, Marina Messetti

**Affiliations:** 1 Department of Psychology, University of Milano-Bicocca, 20126 Milan, Italy; 2 Independent Researcher, 20861 Brugherio, Italy; messetti.marina@gmail.com

**Keywords:** emotion regulation, peer relationships, social acceptance, social rejection, emotional–behavioral problems

## Abstract

Background/Objectives: Children showing emotional–behavioral problems experience lower psychosocial well–being concurrently and in later stages. Developmental research suggests that emotion regulation abilities and the quality of peer relationships play a central role in predicting several behavioral and emotional difficulties. The present study investigates the way emotion regulation skills and peer acceptance and rejection contribute to behavior problems in a sample of Italian schoolers, also verifying the role of gender. Methods: The participants were 220 children (97 boys) aged between 7 and 9 years attending four primary schools in northern Italy. The level of social acceptance and rejection of each child was measured using the sociometric technique of Peer Nomination. In addition, the Emotion Regulation Checklist filled in by the teachers, and the Child Behavior Checklist, completed by the parents, were used to assess children’s emotion regulation abilities and the presence of behavior difficulties, respectively. Results: The main results confirm that behavioral problems are both negatively associated with emotional regulation skills and positively with the social rejection level. However, the impact of peer rejection on the manifestation of problem behavior is fully mediated by children’s ability to regulate their emotions. Interestingly, this pattern of interrelationships only applies to male participants. Conclusions: The study helps to clarify the mechanism through which the degree of peer rejection influences emotional–behavioral difficulties and emphasizes the importance of considering gender-specific processes within interpersonal risk models of problem behavior.

## 1. Introduction

Social relationships with peers are crucial for children’s development, as they provide opportunities to acquire and develop many emotional, cognitive, and social skills [1]. At the same time, the behaviors children engage in in these relationships contribute substantially to determining their quality. For this reason, peers can have an enduring influence on children’s well-being and mental health, broadly defined as the timely achievement of developmental milestones, healthy social and emotional development, and effective regulatory and coping skills [2,3].

The emotional and behavioral problems that can arise in an individual’s interaction with his/her social and relational environment are multifaceted; however, psychopathology identifies internalizing and externalizing problems as the two broadest categories that encompass these types of impairments occurring in childhood [4,5,6].

The definition of externalizing disorders includes behaviors deemed harmful and destructive to others, such as impulsivity, hostility, outbursts of anger and aggression, and hyperarousal [7]. Psychological research has shown that externalizing problems are highly stable throughout development and are associated with long-term negative outcomes, such as criminal and deviant behavior [8,9,10,11]. Conversely, internalizing problems are characterized by negative thoughts and emotions that individuals direct at themselves and generate pain in the form of self-punishment [12]. These include feelings of guilt, excessive worry, a widespread sense of loneliness, social withdrawal, and somatic complaints [13,14,15]. In contrast to externalizing disorders, the literature on the stability of internalizing problems shows less consistent results; while some studies have not found the presence of any continuity of these disorders over time [16], others have shown the presence of predictive relationships between the internalizing problems manifested by children and anxiety-type disorders appearing at later phases [1,9,17].

The prevalence of mental health problems in children and young people living in Europe is quite high: the World Health Organization [18] has certified that one in seven is affected, with anxiety, depression, and behavioral disorders among the most common. These seem to be detrimental to the functioning of the individual, impairing, firstly, the ability to succeed at school and academically and, subsequently, in the work environment [19,20,21].

To safeguard individual and collective psychological well-being, it is, therefore, strictly necessary to identify the risk factors that can lead to the onset of these disorders. To this end, several studies have identified the degree of social rejection that a child experiences, especially in the school context, as one of the factors that may help explain the appearance of these disorders during development. As Baumeister and Leary argued [22], being included and welcomed by one’s reference group generates a wide range of positive emotions, such as joy and euphoria, while, on the contrary, being rejected, excluded, or ignored generates strong negative feelings, such as anxiety, depression, and loneliness. The authors, therefore, emphasize the importance of satisfying the need for social belonging, understood as the need to form and maintain gratifying interpersonal relationships, for the physical and mental health and well-being of individuals. In this regard, research shows that social rejection is associated with the onset of anxiety and depressive symptoms, both in the concurrent stages, as supported by the Reijntjes and colleagues’ study observing 240 children between the ages of 10 and 13 [23], and in the later stages, as shown by, among others, Panak and Garber, who demonstrated that social rejection predicts depressive symptoms one year later [24,25,26]. Difficult relationships with peers also predict the onset of conduct disorders [27], delinquency, and school dropout [28,29]. Furthermore, social exclusion, which is a pervasive aspect of an individual’s life, can lead to, and even exacerbate, the emergence of antisocial behavior in those children who are initially predisposed to aggressive acts [30], as well as experiences of chronic victimization in childhood can foster the subsequent development of anxiety and depression [31].

On the other hand, another strand of studies has investigated the weight and role of some weaknesses in behavioral functioning in determining the presence of possible critical peer relations. Indeed, children who exhibit problematic behavior in the classroom, such as hyperactivity and internalizing and externalizing behavior, typically experience increased interpersonal difficulties that could lead to peer rejection [32]. The landmark studies in this field come from the work of Coie and Dodge and that of Newcomb and colleagues [33,34], who emphasized that the most stable correlates of social rejection are precisely the destructive and aggressive behaviors enacted by children. The authors also indicated that social withdrawal and shyness behaviors, which, as already mentioned, fall into the category of internalizing problems, are associated with lower levels of social acceptance by peers. In fact, from childhood, children who display anxious or depressive behavior experience more negative responses from peers, tend to be disliked and excluded, and suffer victimization [35,36,37]. Therefore, over the years, studies showed the link between emotional–behavioral problems and the level of acceptance and social rejection among peers and underlined the presence of predictive relationships between these two constructs in both directions, helping to highlight the bi-directional link between them.

One of the factors closely associated with social rejection is emotion regulation, which, according to the literature, is also correlated with emotional–behavioral problems. Indeed, emotion regulation enables children to respond to environmental demands by implementing a series of socially acceptable and sufficiently flexible responses [38], thus leading to peer acceptance [39,40,41]. In other words, the ability to regulate one’s internal states is a key ingredient in the processing and choice of contextually appropriate behavior and is involved in the social functioning of every individual [42,43].

The relationship between emotion regulation and psychopathology also appears to be well established in the literature [44,45,46]. The inability to manage emotions, especially negative ones, is strongly associated with the presence of behavioral problems [47,48], so much so that many forms of psychopathology can be considered regulation disorders [49]. In this regard, there is a robust body of empirical evidence that has demonstrated the link between externalization problems and inadequate or deficient forms of emotion regulation [50,51,52]; similarly, emotion regulation problems are shown to be associated with anxiety symptoms, both in children and adults, and precede the onset of depressive symptoms [53,54].

However, the above associations are mainly investigated by considering only two constructs at a time; in fact, only a few studies have attempted to explain how these conceptually distinct but strongly interrelated aspects of development fit into a broader model that includes them all. In this regard, in trying to explain the onset of internalizing problems, namely depression, Fussner and colleagues investigated the influence of emotional dysregulation in mediating the effects that social rejection has on the onset of such emotional–behavioral problems [55]. Assuming that emotion regulation is closely dependent on the social context in which the individual is embedded, rejected children, who are often ignored and excluded, may not have the same opportunities as others to develop appropriate regulation skills [30]. Looking longitudinally at a group of children attending primary school, the authors have highlighted that social rejection, assessed by filling in a questionnaire by teachers, predicts the subsequent onset of depressive symptoms. Moreover, the results led to the assumption that emotion regulation ability, whose development depends on the level of social rejection a child undergoes, mediated the relation of peer rejection to depressive symptoms, but only for boys. Overall, the study provides important insights about emotion dysregulation as a mechanism explaining the relationship between peer rejection and behavioral functioning, underlining the importance of considering the impact of gender in studying the dynamics determining the appearance of childhood emotional–behavioral problems.

Another study investigating how emotion regulation and social rejection explain the onset of behavioral problems is that of Trentacosta and Shaw [56]. Unlike the previous one, the authors tested a model in which emotion regulation impacts social rejection that, in turn, can explain the emergence of externalizing problems, specifically in terms of antisocial behavior. The authors observed a group of children from preschool to adolescence, assessing their regulatory skills through an experimental task, the level of social rejection using the peer nomination technique, and any behavioral problems through reports filled out by the children themselves, their parents, and their teachers. What emerged was that social rejection is directly linked to the capacity for emotion regulation and can explain the indirect effects this last variable has on the appearance of behavioral problems, thanks to its role as a mediator. Therefore, the few studies investigating how social rejection and emotional regulation, taken together, affect children’s emotional–behavioral functioning seem to provide quite inconsistent results. For this reason, further research is necessary to clarify which mechanisms and processes lead to the emergence of behavioral problems, the adverse developmental outcomes of which are well known.

Based on these considerations and adopting the perspective supported by those longitudinal and meta-analysis studies showing significant emotion regulation and peer influence effects for different behavioral outcomes, including externalizing and internalizing problems [4,57], the present study was conducted to simultaneously consider emotion regulation skills and the degree of social acceptance and rejection among peers when examining emotional–behavioral difficulties in a group of school-age children. Furthermore, although the literature testifies to substantial gender differences, both in the children’s emotional regulatory abilities and concerning the onset of emotional–behavioral problems [58,59], only a few studies have examined the pattern of interrelationships between these constructs separately in males and females to highlight any similarities and differences.

In light of these premises, the present study aims to investigate:

1. The direct relationships between emotional regulation, social acceptance and rejection, and behavioral problems, considering the possible impact of gender on their pattern;
2. How emotional regulation and social acceptance and rejection, taken together, contribute to explaining emotional–behavioral problems, also verifying whether these interrelationships assume the same characteristics in males and females.

These objectives were addressed through a multi-method and multi-informant study that envisaged the involvement of the children, their parents, and their teachers.

## 2. Materials and Methods

### 2.1. Participants

The participants in the study were typically developing children aged between 7 and 9 years attending the second and third grades of 4 different schools located in northern Italy.

After presenting the research project to the headmasters, teachers, and parents and obtaining their approval, we delivered the informed consent form to all parents.

Overall, 14 classes agreed to participate in the study, 6 in the second grade and 8 in the third. Excluding those for whom a certified diagnosis of a developmental disorder was attested (N = 5), 220 children took part in the study, of whom 89 (40.45%) attended the second year and 131 (59.55%) the third, with a total of 97 males (44.1%) and 123 females (55.9%) with an average age of 8.4 years (range = 7.17–9.04 years; SD = 7.37 months).

The parents of the children and their teachers, about two per class for a total of 25, actively took part in the study by filling in the questionnaires addressed to them.

The study met ethical guidelines for human subject protection, including adherence to the legal requirements of Italy, and received formal approval from the local Research Ethical Committee of the University of Milano—Bicocca.

### 2.2. Measures

The participants were assessed using different methods of data collection: emotional regulation skills and any emotional–behavioral problems were measured through indirect observation, whereas the degree of social acceptance and rejection among peers was measured using a sociometric technique. All instruments are described below.

#### 2.2.1. Emotion Regulation Checklist

To measure children’s emotional regulation ability, teachers were asked to fill in the Italian version of the Emotion Regulation Checklist (ERC-I) [60]. The other report questionnaire consists of 24 items describing emotionally connoted behaviors, each rated on a 4-point Likert scale (from 1 = almost never to 4 = almost always). It investigates two main dimensions: the first, Emotional Regulation, is measured by 8 items referring to emotional self-awareness, empathy, and the capacity to adjust one’s arousal to adapt to the environment; the Lability/Negativity subscale, instead, comprises 16 items assessing inflexibility, dysregulated negative affect, and unpredictability and suddenness of mood change.

The validation study of the ERC-I for teachers [60] supports the structure validity and reliability of the instrument. The Confirmatory Factor Analysis results indicated a Root Mean Square Error of Approximation (RMSEA) of 0.072 and a Comparative Fit Index (CFI) of 0.98. The Cronbach’s alpha values are 0.73 for the Emotional Regulation and 0.89 for the Lability/Negativity subscales, respectively.

For each child, a total Emotional Regulation index (range 24–96) was calculated by summing the raw scores obtained in the two dimensions (for this purpose, the values of the Lability/Negativity subscale are to be considered inversely), and then converting them into z-scores. Higher scores indicate a greater capacity to manage and modulate one’s emotional arousal, thus fostering an appropriate level of engagement with the environment.

#### 2.2.2. Child Behavior Checklist

The Child Behavior Checklist (CBCL) is a widely used instrument to examine adaptive behavior and identify behavioral and emotional problems in children and adolescents aged 6 to 18 [61].

The questionnaire, completed by the parents, is divided into two scales. The first provides information on the child’s social competence through 20 items that investigate his/her degree of participation in various sports, home, and school activities, as well as the quality of his/her relationships with peers, siblings, and parents. The second scale, the only one considered for the present study, consists of 113 questions to be answered on a three-level Likert scale (0 = not true, 1 = sometimes true, 2 = very true) and examines the child’s behavior in the present and the previous 6 months. In more detail, this problem behavior scale is structured on eight syndromic subscales grouped into two higher-order factors, internalizing and externalizing problems, and a third dimension generically called other problems. Internalizing problems refer to anxious, depressive, and somatic complaints, whereas externalizing problems include rule-breaking and aggressive behaviors; moreover, the other problems dimension covers social, attentional, thought-related, and other problems that do not fall under the difficulties described above.

In this study, we used the Italian version of the instrument [62], whose structural validity was evaluated with Confirmatory Factor Analysis that showed a RMSEA of 0.076 and a CFI of 0.977. Additionally, the values of Cronbach’s alpha were 0.87 for the internalization dimension and 0.86 for the externalization dimension, respectively.

Since the standardized scores have an exclusively clinical–diagnostic purpose, the raw scores obtained for each of the three dimensions were used in this study. Thus, the score for Internalization problems, ranging from 0 to 64, the score for Externalization problems, ranging from 0 to 70, and the score for Other problems, ranging from 0 to 106, were considered. The sum of these scores provides a further index, called Total problems, ranging from 0 to 240, where higher scores correspond to a greater presence of problem behavior.

#### 2.2.3. Peer Nomination Technique

Children’s degree of social acceptance and rejection by classroom peers was measured using the sociometric technique of peer nomination [63]. This technique is designed to measure the interplay of attractions and repulsions that individuals in a group manifest toward one another by asking each participant to express a limited number (usually three, as in the present study) of preferences and non-preferences toward the other members of the reference group. Generally, the number of sociometric criteria (i.e., the questions on a sociometric test) varies between two and four, a number considered sufficient to obtain adequate information [64].

In this study, children were presented with the following questions to answer in written form:

- Of your classmates, who would you like to have as your deskmate?
- Of your classmates, who would you not like to have as a deskmate?
- You go on a school trip with your class and teachers. Which classmate would you like to stay with and do the activities proposed during the day?
- You go on a school trip with your class and teachers. Which classmate would you not like to stay with and do the activities proposed during the day?
- Which classmate would you like to do your homework with?
- Which classmate would you not like to do homework with?

The questions are selected to obtain social acceptance and rejection scores that reflect choices based on both personal criteria (i.e., affinity and liking) and social criteria (i.e., children’s abilities and aptitudes that may be useful for the achievement of a specific objective); moreover, they refer both to the school environment and circumstances outside the school.

The following measures were considered for each participant: the social acceptance index (i.e., the ratio of the absolute frequency of positive choices received to the total number of choices each child could have received) and the social rejection index (i.e., the ratio of the absolute frequency of rejections received to the total number of rejections each child could have received), referring to the degree to which an individual is liked or disliked by peers. The two indices (scoring between 0 and 1) were standardized to control for the possible effect of school class numerosity.

### 2.3. Statistical Analyses

Data analyses were conducted using IBM SPSS (Statistical Package for the Social Sciences) software, version 29.0.1.0.

To examine the data distribution, skewness and kurtosis analyses were conducted. The results showed that some of the variables considered (i.e., CBCL Externalizing problems, Other problems, and Total problems scores) were not normally distributed according to the criteria proposed by George and Mallery, which require skewness and kurtosis values between −2 and +2 [65]. Therefore, the relative scores were transformed using a logarithmic function with base 10 to use parametric tests.

As a preliminary step, correlational analyses were conducted to check for possible relationships between the age of the participants and the observed variables; moreover, a series of *t*-tests were carried out to examine any gender differences in the same measures. Subsequently, direct relationships between the variables of interest were analyzed using correlational analyses. Additionally, to explore any indirect relationship between emotional regulation abilities, peer rejection degree, and emotional–behavioral difficulties, bootstrapped mediation analyses (with 5000 bootstrap samples) were conducted. Tests were bilateral, with a statistical significance set at 0.05.

## 3. Results

### 3.1. Age and Gender Influence

The descriptive statistics of the observed variables for all participants and for males and females separately are shown in Table 1.

To first verify the presence of any relationships between the age of the participants (given its degree of variability) and the outcome variables, Pearson correlational analyses were carried out, none of which led to significant results from a statistical point of view.

Secondly, starting from the results reported in the literature showing different social–emotional and behavioral functioning between males and females, a series of *t*-tests for independent samples were conducted to investigate the presence of such discrepancies in the present study’s group of participants. The results obtained testify to multiple differences according to children’s gender. First, it is confirmed that females appear less rejected (t _(218)_ = 3.590; *p* < 0.001; Cohen’s d = 0.487) and tend to be more accepted (t _(218)_ = −1.955; *p* = 0.052; Cohen’s d = −0.265) than their male companions; furthermore, male children, in addition to being less able to regulate their emotions (t _(218)_ = −4.871; *p* < 0.001; Cohen’s d = −0.661), obtain higher scores in both externalizing (t _(193)_ = 2.322; *p* = 0.021; Cohen’s d = 0.335) and other problems (t _(193)_ = 2.678; *p* = 0.008; Cohen’s d = 0.386), as well as in the total problems index of CBCL, which summarizes the behavioral functioning of children in problematic terms (t _(193)_ = 2.278; *p* = 0.024; Cohen’s d = 0.329). No statistically significant differences emerged concerning the internalizing behaviors score.

### 3.2. Direct Relationships Between Emotion Regulation Skills, Peer Acceptance and Rejection, and Emotional–Behavioral Problems

Given the numerous gender differences that emerged in the variables considered and to check whether the patterns of relationships between them are comparable or differ according to the gender of the children, correlational analyses were conducted separately for boys and girls.

As Table 2 shows, male participants who, according to their teachers, are less able to regulate emotions obtain higher scores both in the subscale measuring problematic externalizing behavior and in the subscale that assesses problems related to other syndromes that do not fall into the classic internalizing/externalizing problem subdivision; there is also a negative statistically significant correlation between the score these children obtained on the ERC and the total recorded in the CBCL. On the contrary, no statistically significant correlations were found for the female participants.

As regards the association between behavioral problems and social outcomes too, numerous relationships emerged exclusively in the group of male participants, emphasizing that more behaviorally problematic children face less acceptance and increased social rejection by members of their reference group; there were, indeed, statistically significant correlations between the total score obtained on the CBCL and those on social acceptance and rejection, the former negative and the latter positive.

More specifically, the degree of social acceptance negatively correlates with the score on both externalizing and other behavioral problems. Furthermore, when considering the level of social rejection, it appears that this correlates positively and statistically significantly with all the indices relating to the subscales of the CBCL (internalizing, externalizing, and other problems).

### 3.3. Indirect Relationships Between Emotion Regulation Skills, Peer Acceptance and Rejection, and Emotional–Behavioral Problems

The previous correlational analyses show that behavioral problems are significantly related to social outcomes and emotional regulatory capacities, exclusively in male children. Consequently, to examine in more depth the influence of both these factors, not only taken individually but also jointly, a mediation analysis was conducted.

For this purpose, the relationships between emotional regulation and social acceptance and rejection were first tested, limited to male participants. The correlation coefficients show that higher emotional regulation scores correspond to higher social acceptance (r = 0.386; *p* < 0.001) and lower social rejection ratings (r = −0.560; *p* < 0.001).

**Figure 1:**
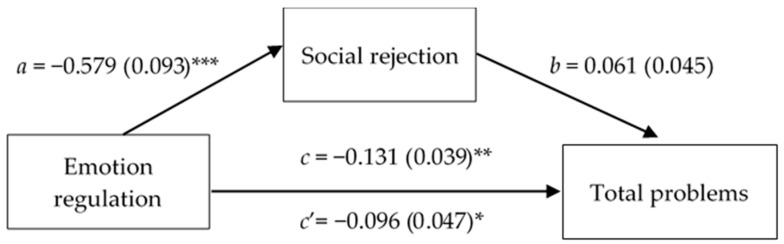
Simple mediation diagram: *a*, *b*, *c*, and *c’* are path coefficients representing unstandardized regression weights and standard errors (in parentheses). The *c* path coefficient represents the total effect of the emotion regulation index on the CBCL total problems score. The *c’* path coefficient refers to the direct effect of the emotion regulation index on the CBCL total problems score. * *p* < 0.05; ** *p* < 0.01; *** *p* < 0.001.

In the face of such results, using the macro process of Preacher and Hayes [66], a first analysis of mediation was carried out with the emotion regulation ability included as the independent variable, social rejection as the mediator, and emotional–behavioral problems, operationalized as the CBCL total score, as the output variable. The results (see Figure 1) demonstrate that emotional regulation has a total (β = −0.131; SE = 0.039; *p* = 0.001; CI = −0.208, −0.054) and direct (β = −0.096; SE = 0.047; *p* = 0.042; CI = −0.188, −0.003) effect on emotional–behavioral problems that are statistically significant; conversely, the indirect effect is not substantial (β = −0.035; SE = 0.024; CI = −0.081, 0.017) since emotional regulation predicts social rejection (β = −0.579; SE = 0.093; *p* < 0.001; CI = −0.764, −0.394), but the latter does not have a statistically significant impact on emotional and behavioral difficulties, excluding the contribution of the emotion regulation factor (β = 0.061; SE = 0.045; *p* = 180; CI = −0.029, 0.151).

**Figure 2:**
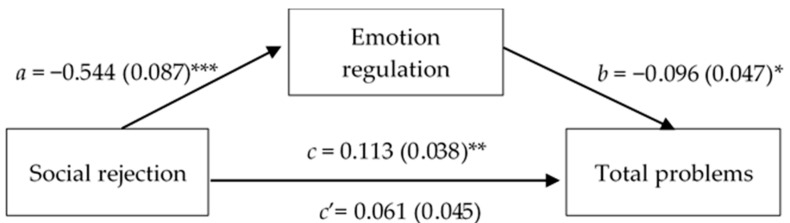
Simple mediation diagram: *a*, *b*, *c*, and *c’* are path coefficients representing unstandardized regression weights and standard errors (in parentheses). The *c* path coefficient represents the total effect of the social rejection index on the CBCL total problems score. The *c’* path coefficient refers to the direct effect of the social rejection index on the CBCL total problems score. * *p* < 0.05; ** *p* < 0.01; *** *p* < 0.001.

Further mediation analysis was then conducted to sustain the hypothesis that emotion regulation mediates the effects of social rejection on emotional–behavioral problems (Figure 2). The results show that the impact of social rejection on problem behavior is fully mediated by emotional regulation capacity, as evidenced by a statistically significant total effect (β = 0.113; SE = 0.038; *p* = 0.004; CI = 0.037, 0.189) alongside a statistically non-significant direct effect (β = 0.061; SE = 0.045; *p* = 0.180; CI = −0.029, 0.151).

## 4. Discussion

This study aimed to investigate whether and how the emotional–behavioral problems manifested by the children relate to their emotional regulation ability, as well as correlate with the degree of social acceptance and rejection by peers.

Overall, the pattern of results concerning a group of children between the ages of 7 and 9 testifies to several direct relationships between the constructs examined; nevertheless, these relationships seem to relate exclusively to the male gender.

First of all, it emerges that the behavioral problems of boys, excluding the internalizing ones, are negatively associated with their emotional regulatory abilities, even if these difficulties do not assume a clinically relevant dimension, as in the participants observed in this study. This result is consistent with the hypothesis, as reported in the literature, that various forms of psychopathology, including conduct disorders, oppositional defiant ones, and ADHD, can be considered real regulation disorders given that the ability to effectively manage and respond to emotional experiences plays a central role in children’s psychological functioning [49,67]. In light of this consideration, it becomes equally relevant that the male participants examined in this study not only show more dysfunctional behavior than their female peers but are also characterized by lower emotion regulation. These gender discrepancies are supported by studies indicating that boys react in a dysregulated manner when faced with unpleasant or stressful situations and, at the same time, display direct aggressive behavior more frequently than girls [68,69].

As previous research testifies [39,70], failing to modulate emotions can be considered a risk factor for being rejected by peers as cascading effects were found from emotion regulation abilities that children exhibit at preschool age to social skills a few years later which, in turn, are related to greater peer acceptance and greater emotion regulation observed in middle childhood [41]. According to these findings, this study shows, limited to boys, that the ability to regulate one’s emotions is concurrently related to the degree of both peer rejection and acceptance. So, good emotional regulation skills can improve children’s ability to engage in positive social relationships with peers, whereas, in contrast, greater challenges in coping and emotional control can increase the probability of being exposed to adverse peer experiences, such as social exclusion [42,71,72]. This consideration also seems to be, in part, supported by the fact that the girls who participated in this study, in addition to demonstrating more improved emotional regulation skills, also had significantly lower peer rejection scores and a higher degree of social acceptance.

Overall, these findings corroborated the idea that emotional regulation skills play an important role both in fostering positive relationships with peers and promoting children’s adaptation through adequate behavioral functioning. Nevertheless, although once again only in the male group, behavioral functioning too appears to be directly related to social outcomes, as evidenced by the finding that children who are more rejected by peers and experience less social acceptance more frequently display problematic behaviors, both internalizing and externalizing, as well as other problems measured by the CBCL. These results support existing evidence showing that the behaviors children act within social relationships contribute substantially to determining their quality (in terms of social acceptance and rejection by peers); at the same time, dysfunctional peer relationships, which result in social rejection, exclusion, and isolation, contribute to the onset of internalizing and externalizing disorders over time [27,73]. In other words, especially during middle childhood, as peer relationships become more meaningful, they can play a crucial role in several areas of functioning, including behavioral and emotional development [23].

Starting from the outlined relationships and considering the negative impact that emotional–behavioral difficulties have on an individual’s personal and social functioning, it is crucial to examine how emotional regulation ability and peer rejection, jointly considered, contribute to potentially determining behavioral dysfunction. The hypothesis that emotional regulation ability influences children’s behavioral functioning via the degree to which an individual is disliked by peers seems to be excluded. In fact, no effect of the mediating variable, namely social rejection, was observed on the level of problem behavior manifested by children. Although a direct relationship has been found between social rejection and behavioral difficulties, this link disappears if the contribution of emotional regulation skills is simultaneously considered. This result seems to underline the importance of the regulatory component as the core of the associations between the constructs investigated. This consideration is supported by the results deriving from the second analysis of mediation conducted, indicating that social rejection constitutes a risk factor for the appearance of behavioral problems only if associated with poor regulatory skills. In other words, according to the results reported by Fussner and colleagues [55], social rejection could be accompanied by unfavorable conditions for adequate development of regulatory skills that, in turn, could contribute to generating behavioral problems.

Overall, this study helps to support the importance of social processes, particularly peer relationships, in leading to negative developmental outcomes. At the same time, however, it provides support for the role of emotion regulation skills as mediators between peer rejection and the presence of dysfunctional behavior. On the other hand, this model is well suited to represent the interactions between the investigated constructs as they occur in male participants but does not adequately represent the links between the same variables in females. In this regard, it has to be noted that empirical evidence suggests that girls are not only more likely than boys to engage in adaptive coping in response to peer-related stress but also that boys and girls may experience different types of peer rejection which, in turn, may generate distinct patterns of emotion dysregulation [74,75]. Consequently, the relationship between peer rejection and emotion regulation may vary depending on the children’s gender, influencing the role of emotional skills on the problem behavior observed in the female participants. In light of these considerations, it becomes important to consider gender-specific processes within interpersonal risk models of problem behavior to further clarify the potential moderating effect of this variable in the multifaced interplay between emotion regulation skills, peer relationships, and less adaptive behavior.

In conclusion, this study hints at some important clinical implications for the prevention and treatment of emotional–behavioral problems. Indeed, over the past few years, numerous intervention programs have been developed to reduce social rejection and teach children effective strategies for managing conflict and learning to interact appropriately with peers. However, the interventions could be strengthened by focusing on the effects of social rejection in terms of emotional dysregulation; in particular, helping children to develop adaptive strategies for emotion regulation could mitigate the negative consequences of peer rejection on the onset of internalizing and externalizing behavioral problems.

It, therefore, seems important to promote the development of these capacities early on in education through targeted programs that not only consider children’s developmental stage but also gender differences.

This study has some limitations that deserve to be acknowledged. The cross-sectional design adopted does not make it possible to establish a clear cause–effect relationship between the factors examined. Although it is plausible, also in light of the reported literature, that the ability to regulate emotions plays a crucial role in the development of both positive peer relationships and adaptive behaviors and attitudes, only a longitudinal investigation would make it possible to exclude the equally acceptable hypothesis that any emotional–behavioral difficulties may result in a greater rejection by peers, exacerbating the early fragilities.

Additionally, the proposed mediation model includes, as the only outcome variable, the total CBCL score, which provides information on the child’s overall level of functioning. Further research is certainly needed to investigate the potentially different roles the factors examined may play when considering internalizing and externalizing problems separately and to identify the multiple pathways linking these problems with peer relationship experiences and the development of specific social–emotional skills.

## Figures and Tables

**Table 1:** Mean and standard deviation of the observed variables.

	All Participants			Males			Females		
	N	M	SD	N	M	SD	N	M	SD
Social acceptance	220	0.14	0.09	97	0.13	0.09	123	0.15	0.09
Social rejection	220	0.12	0.13	97	0.15	0.15	123	0.09	0.11
Emotion regulation	220	80.64	11.56	97	76.48	13.35	123	83.91	8.66
Internalizing problems	195	5.76	5.17	86	5.97	4.96	109	5.60	5.36
Externalizing problems	195	4.59	4.33	86	5.49	5.06	109	3.89	3.51
Other problems	195	9.54	7.87	86	11.43	9.03	109	8.05	6.48
Total problems	195	19.89	15.71	86	22.87	17.58	109	17.53	13.69

**Table 2:** Pearson correlation indices between emotion regulation, peer social acceptance and rejection, and behavioral problems scores.

	Emotion Regulation		Social Acceptance		Social Rejection	
	Males	Females	Males	Females	Males	Females
Internalizing problems	−0.176	0.007	−0.198	−0.033	0.233 *	0.007
Externalizing problems	−0.372 ***	−0.052	−0.307 **	0.036	0.365 ***	−0.005
Other problems	−0.357 ***	0.066	−0.223 *	0.034	0.309 **	−0.021
Total problems	−0.347 ***	0.026	−0.223 *	0.039	0.309 **	0.000

* *p* < 0.05; ** *p* < 0.01; *** *p* < 0.001.

## Data Availability

The data presented in this study are available on request from the corresponding author. The data are not publicly available due to ethical and GDPR reasons.
